# Blood Lead Levels and Cardiovascular Disease Risk: Results from the Korean National Health and Nutrition Examination Survey

**DOI:** 10.3390/ijerph181910315

**Published:** 2021-09-30

**Authors:** Yoonjin Park, Jungjin Han

**Affiliations:** 1 Department of Nursing, Joongbu University, Geumsan 32716, Korea; pyj2272@naver.com; 2 Department of Nursing, Semyung University, Jecheon 27136, Korea

**Keywords:** lead, cardiovascular disease, Framingham risk score, heavy metals, health promotions, community health

## Abstract

(1) Objective: Lead, a heavy metal that exists commonly in air, soil and crops may cause chronic disease in the cardiovascular system. The purpose of this study is to investigate how blood lead levels affect cardiovascular disease in adults. (2) Study Design and Participants: It is a cross-sectional, descriptive study using data from the Korean National Health and Nutrition Examination Survey (KNHANES). Data from a total of 1929 participants, derived from the KNHANES, conducted by the Korea Centers for Disease Control and Prevention, in 2017, were analyzed using SPSS version 25.0. (3) Measurement: The cardiovascular disease risk was calculated using the Framingham risk score. There was a strong positive correlation between blood lead levels and the Framingham risk score. Furthermore, of the FRS sub-criteria, systolic blood pressure, HDL cholesterol level and total cholesterol level all also showed a significant correlation. (4) Results: We analyzed the correlation between PbB levels and the FRS sub-criteria, including systolic blood pressure, HDL cholesterol level, total cholesterol level and the FRS total. We found a significant positive correlation between PbB levels and systolic blood pressure, FRS total and total cholesterol level (*p* < 0.05), as well as a significant negative correlation with HDL cholesterol level (*p* < 0.05). (5) Conclusion: Based on the perception that there is no lower toxicological threshold for blood lead, it is necessary to restrict lead in product manufacturing for the purpose of public health. In addition, it is necessary to be aware of the dangers of exposure to even small amounts of lead in daily life.

## 1. Introduction

The U.N.’s sustainable development goal (SDG) is to minimize the pollution of air, water and soil by chemicals by 2030. Lead (Pb) is a heavy metal that was ranked second among the major toxic substances in 2007 by the United States of America Agency for Toxic Substances and Disease Registry (ATSDR), and a priority heavy metal handled by the Ministry of Food and Drug Safety [1]. Since Pb is a heavy metal that is found widely in air, soil, exhaust gases, crops and cosmetics, it must be efficaciously managed.

According to the Toxicological Profile on Lead by the Agency for Toxic Substances and Disease Registry, a blood Pb (PbB) concentration of 10µg/dL is the general standard of risk. However, a number of recent studies have reported that the mortality rate increases even at lower PbB levels [2,3,4].

Generally, Pb enters the human body via ingestion. However, Pb can also enter the human body via inhalation or skin contact. Pb can adversely affect the respiratory, cardiovascular, musculoskeletal, renal and nervous systems and lead to cancer [5]. Pb has a half-life of about 30 days in human blood. However, Pb can accumulate over several decades in the cortical bone. This accumulated Pb can then be released back into the blood owing to pregnancy, lactation, menopause, weight loss, or osteoporosis [1,6]. The risk of direct and indirect health effects increases with age. Additionally, PbB interferes with inhibition of delta-aminolevulinic acid dehydratase enzyme, erythrocyte formation, decreases the erythrocyte survival time and increases the risk of anemia by inhibiting hemoglobin production [7,8]. Furthermore, chronic Pb poisoning can result in vascular endothelial tissue damage, blood coagulation dysfunction and increased platelet levels, which can induce cardiovascular disease (CVD) [9].

Although it is challenging to establish a clear association between PbB and CVD due to multiple confounding factors such as age, obesity, ethnicity, smoking and drinking, several previous studies have reported the occurrence of CVD-related conditions including hypertension, atherosclerosis and cardiac conduction disorder as well as increased mortality with long-term Pb exposure. Evaluations of bone Pb levels, which build up over a long period of time, have shown that long-term Pb exposure, even at low levels, can cause ventricular myocardial and cardiac conduction problems [10]. It has also been found that heavy metals such as Pb can cause the hardening of the carotid intima-media thickness [11]. Additionally, a long-term prospective study of male veterans reported that the group with higher bone Pb and PbB levels had a higher incidence of ischemic heart disease [12], demonstrating the association between long-term Pb exposure and CVD risk.

There are various methods to predict CVD risk. A method that is used widely is the Framingham risk score (FRS). The FRS is a way of calculating and predicting the 10-year CVD risk by quantifying the selected major risk factors of CVD such as sex, age, blood pressure, total cholesterol level, high-density lipoprotein (HDL) level, smoking status, use of antihypertensives and diabetes [13].

In this study, we used the Korean National Health and Nutrition Examination Survey (KNHANES) data to analyze PbB levels and CVD risk using the FRS in the Korean population.

## 2. Materials and Methods

### 2.1. Study Design and Participants

This study is a cross-sectional descriptive study using secondary data from KNHANES phase VII-1 (2017) conducted by the Korea Center for Disease Control and Prevention. The KNHANES has been conducted annually by the Korea Disease Control and Prevention Agency (KCDC) since 2007 and includes a health examination and interview in a mobile unit. Twenty-three households in 192 districts are chosen every year as a probabilistic sample, with approximately 10,000 household members aged over 1 year surveyed.

**Figure 1:**
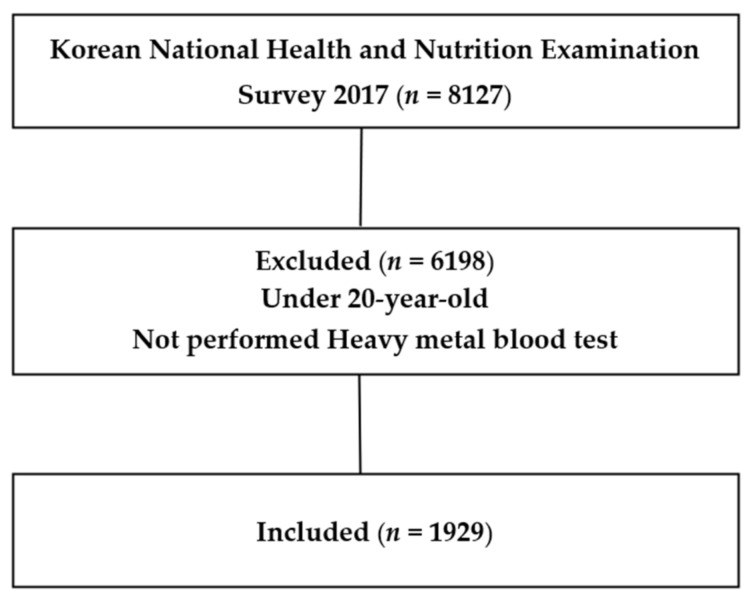
Simplified flow chart of study subject selection.

Multi-stage, stratified, cluster sampling, which is a complex sample design method, was used to extract samples from KNHANES data for representativeness and improved accuracy of estimation. This is a step-by-step cluster extraction by selecting the first sample based on the cluster, selecting the second sample from the selected cluster, and extracting the third sample, unlike in the case of simple random extraction [14]. A total of 10,430 individuals were included in Year 2 of the 7th KNHANES (2017), and 8127 individuals (participation rate of 77.9%) participated in more than one of the surveys, encompassing a health survey, screening survey and nutrition survey. In 2017 KNHANES, heavy metal blood test was not conducted for all participants. Instead, two to three participants were randomly selected for the test according to their enumerated district, sex and age. The participants of our current study were 1929 adults, over the age of 20, who underwent a heavy metal blood test (Figure 1).

### 2.2. Measures

#### 2.2.1. Demographic and Clinical Characteristics

We collected sex, income level, education level, smoking status and body mass index (BMI) information from the participants. Real income per household unit was assessed to estimate the average household income, which was first classified into 30%, and 40% of the total. The upper level of household income was divided into a highest and upper middle group. Lower levels were further subdivided into a lowest and lower middle group. The level of education was classified as ≤6 years, 7–9 years, 10–12 years, and ≥13 years. Smoking status was divided into currently smoking and non-smoking status.

#### 2.2.2. Biochemical Measurements

For the blood test, samples were collected after at least 8 h of fasting, mostly using the median cubital and cephalic veins. Blood samples were refrigerated and then transported to a clinical laboratory on the same day to perform the analysis within 24 h. Triglyceride, HDL and fasting glucose were measured via enzymatic methods using the automatic analyzer 7600 (Hitachi LtD, Tokyo, Japan). Heavy metals were analyzed via Automic Absorption Spectrophotometry using the Perkin Elmer AAnalyst 600.

#### 2.2.3. Cardiovascular Risk

For the CVD risk score, we used the FRS revised in 2008. This calculation divided the participant’s age into nine groups, total cholesterol levels, high-density lipoprotein cholesterol levels, systolic blood pressure and diastolic blood pressure into five groups, and each group was given a unique risk score according to gender. Participants’ current smoking ability and diabetes were divided into two groups according to their presence or absence, and unique risk scores were given according to gender. In this way, the FRS was calculated through the sum of each score estimated in the six processes, and the 10-year coronary heart disease risk was estimated based on the score. We referred to the scoring system used in a previous study and defined a calculated risk of <10%, 10–19% and ≥20% as low, moderate and high risk, respectively [13].

#### 2.2.4. Data Analysis

We used SPSS version 25.0 (IBM, Seoul, Korea) for the statistical analysis. The enumeration of districts of KNHANES included in the analysis were affected using a multi-stage, stratified, cluster sampling method, which is a complex sample design method. A complex sample analysis method of SPSS was used to add sample weights for analysis. The sample weights of KNHANES are a raising multiplier applied so that the estimated value is representative of the entire Korean population and are calculated by reflecting the extraction rate, response rate and distribution of the population. Moreover, if a new variable was created by combining several variables or a statistical model that included several variables for analysis was used, investigation categories, areas and items associated with all the variables were considered. In other words, sample weights that included many categories, areas, and items were referred to as correlation analysis weights in the KNHANES, with weights provided for each year.

We performed the Chi-square test and analysis of variance (ANOVA) to analyze differences between groups based on general characteristics and PbB levels. To identify the predictors of CVD risk, we analyzed the FRS sub-criteria, including blood pressure, HDL cholesterol level, total cholesterol level and corresponding PbB level using multinomial logistic regression. The correlation with each variable was verified using Pearson’s correlation coefficient.

#### 2.2.5. Ethical Considerations

KNHANES data was used in this study. It was anonymized such that personal information of the participants cannot be identified and was uploaded onto the website of the Korea Disease Control and Prevention Agency. The study was conducted after being reviewed for exemption by the Institutional Review Board (JIRB-2020021701-02-200218).

## 3. Results

### 3.1. Demographic and Clinical Characteristics

Of the 1929 participants, 894 were male, and 1035 were female. In the low-risk group with an FRS < 10%, males in their 30 s (17.6%) and females in their 50 s (22.8%) were the most prevalent. In the FRS ≥ 20% group, males and females in their 70 s or older were the most prevalent, while age and the FRS were significantly different (*p* < 0.001). Regarding the relationship between income and the FRS, the high-income group was the most prevalent for both males and females in the FRS < 10% group (*p* < 0.001). Regarding education level, individuals with a high level of education ≥ 13 years were the most prevalent in the FRS < 10% group. Contrastingly, in the high-risk group with an FRS ≥ 20%, individuals with an education level < 6 years were the most prevalent. The length of education and FRS displayed significant differences (*p* < 0.001). There was a significant difference between PbB levels and the FRS sub-criteria, including hypertension, diabetes and systolic blood pressure in both males and females (*p* < 0.001). However, HDL and total cholesterol levels were significantly different in females only (*p* < 0.05). Smoking also displayed a significant difference in both males and females (*p* < 0.05). Regarding the relationship between PbB and FRS, in the PbB < 2 µg/dL group for both males and females, an FRS < 10% was the most prevalent and demonstrated a significant between-group difference (*p* < 0.005) (Table 1).

### 3.2. Correlation between FRS Sub-Criteria and the PbB Levels

We analyzed the correlation between PbB levels and the FRS sub-criteria, including systolic blood pressure, HDL cholesterol level, total cholesterol level and the FRS total. We found a significant positive correlation between PbB levels and systolic blood pressure, FRS total and total cholesterol level (*p* < 0.05) as well as a significant negative correlation with HDL cholesterol level (*p* < 0.05) (Table 2).

### 3.3. FRS Predictors

An analysis of the FRS sub-criteria, including systolic blood pressure, total cholesterol level, HD cholesterol level, and PbB level, revealed that in the group with 10–20% CVD risk, the FRS risk predictors were PbB level and systolic blood pressure in males and systolic blood pressure in females (*p* < 0.001). In the group with ≥20% CVD risk, the FRS risk predictors were PbB level, systolic blood pressure and total cholesterol level in males and systolic blood pressure and total cholesterol level in females (*p* < 0.05). In males, the PbB levels were 2.4 times higher with a 10-year CVD risk of 10–20% (OR: 2.407, 95% Cl: 1.885–3.075, *p* < 0.001) and 2.8 times higher with a ≥20% risk (OR: 2.847, 95% Cl: 2.020–4.011, *p* < 0.001), whereas there was no significant difference in females (Table 3).

## 4. Discussion

This is a descriptive study that investigates the association between PbB levels and CVD risk. The results show that a high proportion of those that were younger, had high-income levels and were highly educated belonged to the low-risk group with an FRS of <10%. This is consistent with previous findings that CVD risk increases with age [15] and that socioeconomic status and CVD risk are inversely proportional [16,17].

Regarding the relationship between PbB levels and FRS, the low-risk group with an FRS of ≤10% had low PbB levels below 2 µg/dL in both males and females. Furthermore, of the FRS sub-criteria, systolic blood pressure was significantly low, and HDL was significantly high. This is consistent with previous findings that heavy metals increase blood pressure and blood cholesterol levels [18]. Additionally, it has been found that total cholesterol levels are significantly higher in workers who deal with Pb [19] and that there is a significant negative correlation between PbB and HDL cholesterol levels [20]. In this study, there was a strong positive correlation between PbB levels and the FRS.

Furthermore, of the FRS sub-criteria, systolic blood pressure, HDL cholesterol level and total cholesterol level all showed a significant correlation. This supports the previous findings that PbB levels negatively affect systolic blood pressure, diastolic blood pressure and HDL, increase the risk of metabolic syndrome [21] and is a significant predictor of CVD incidence.

In particular, in the moderate-risk group with an FRS of 10–20% and the high-risk group with an FRS of ≥20%, PbB levels and systolic blood pressure were significant predictors of CVD incidence in males. Moreover, with increasing PbB levels, the 10-year CVD risk increased 2.4 times in the FRS 10–20% group and 2.8 times in the FRS ≥20% group. This is similar to the previous finding that risk of death due to CVD increases with long-term Pb exposure [22] and supports the findings that risk of coronary stenosis increases 1.25-fold with every 1 µg/dL increase in PbB [23] as well as that the risk of CVD death is 2.5 to 5.63 times greater in subjects with higher levels of Pb in the body than normal subjects [6]. However, in females, PbB levels were not a major predictor of CVD risk, unlike in males. This is similar to previous findings that females generally have lower PbB levels than males due to differences in related factors like occupation, drinking, and smoking [20,23,24]. Since more than 90% of PbB exists in erythrocytes, PbB may have more negatively affected the hematological indicators in males, who have a relatively greater number of erythrocytes [7]. However, with recent increases in women’s social activities, drinking, and smoking, the concentration of heavy metals, including Pb, could serve as a critical indicator of CVD incidence and cannot be overlooked even in females.

In the present study, we confirmed a significant correlation between PbB levels and the FRS. This suggests that among the various risk factors of CVD, environmental Pb can be a factor. Therefore, it is necessary to improve the local environmental conditions and increase individual awareness of hazardous substances. We believe that the present findings will serve as a useful indicator of community health.

## 5. Conclusions

In this study, we demonstrated that PbB levels could be used as a major predictor of CVD incidence. Although Pb use in industrial facilities has rapidly decreased with increasing awareness of its adverse effects, it is still present in some toys, cosmetics, food and accessories. Therefore, it can accumulate in our body without occupational exposure. To prevent this, we need a systematic approach involving dietary and environmental improvements, and cooperation among the industrial, environmental and healthcare sectors to improve the national health status. Therefore, we propose the development of a health care program that can prevent CVD and reduce community exposure to heavy metals.

However, the limitation of this study is that the consideration of regional characteristics such as urban and rural, and occupational groups with high and low exposure to lead was not investigated separately.

## Figures and Tables

**Table 1:** Baseline characteristics of participants according to 10-Year CVD Risk (%) (*n* = 1929).

Category	Male (894), *n* (%) or M ± SD				Female (1035), *n* (%) or M ± SD			
	<10%	10–20%	>20%	*p*	<10%	10–20%	>20%	*p*
Age, year								
<30	131 (14.7)	-	-		143 (13.8)	-	-	0.000
30–39	157 (17.6)	-	-		184 (17.8)	-	-	
40–49	146 (16.3)	24 (2.7)	2 (0.2)	0.000	208 (20.1)	-	-	
50–59	97 (10.9)	73 (8.2)	19 (2.1)		236 (22.8)	8 (0.8)	-	
60–69	41 (4.6)	69 (7.7)	24 (2.7)		119 (11.5)	29 (2.8)	2 (0.2)	
≥70	2 (0.2)	49 (5.5)	60 (6.7)		41 (4.0)	56 (5.4)	9 (0.9)	
Household Income, (%)								
Lowest	48 (5.4)	43 (4.8)	38 (4.3)	0.000	113 (10.9)	45 (4.4)	7 (0.7)	0.000
Lower middle	127 (14.2)	49 (5.5)	34 (3.8)		240 (23.2)	26 (2.5)	1 (0.1)	
Upper middle	192 (21.5)	58 (6.5)	14 (1.6)		278 (26.9)	13 (1.3)	1 (0.1)	
Highest	207 (23.2)	64 (7.2)	18 (2.0)		300 (29.0)	8 (0.8)	2 (0.2)	
Educational (years)								
0–6	21 (2.5)	40 (4.7)	36 (4.3)	0.000	125 (12.6)	54 (5.4)	6 (0.6)	0.000
7–9	34 (4.0)	36 (4.3)	16 (1.9)		79 (7.9)	11 (1.1)	-	
10–12	198 (23.4)	75 (8.9)	23 (2.7)		310 (31.2)	21 (2.1)	3 (0.3)	
13 or more	289 (34.1)	57 (6.7)	22 (2.6)		380 (38.2)	4 (0.4)	1 (0.1)	
Hypertension, %				0.000				0.000
No	526 (58.8)	145 (16.2)	42 (4.7)		832 (80.4)	30 (2.9)	1 (0.1)	
Yes	60 (6.7)	76 (8.5)	64 (7.2)		99 (9.6)	63 (6.1)	11 (1.1)	
Diabetes mellitus, %				0.000				0.000
No	542 (60.6)	180 (20.1)	81 (9.1)		873 (84.3)	73 (7.1)	11 (1.1)	
Yes	32 (3.6)	35 (3.9)	24 (2.7)		58 (5.6)	20 (1.9)	-	
Dyslipidemia, %				0.000				0.000
No	514 (57.5)	164 (18.3)	75 (8.4)		782 (75.6)	55 (5.3)	5 (0.5)	
Yes	60 (6.7)	51 (5.7)	30 (3.4)		149 (14.4)	38 (3.7)	6 (0.6)	
BPsys, mmHg	115.92 ± 11.93	123.47 ± 14.50	137.09 ± 14.37	0.000	18.86 ± 13.37	142 ± 16.41	15.82 ± 14.94	0.000
HDL cholesterol (mg/dL)	47.03 ± 10.48	46.63 ± 11.79	46.41 ± 13.67	0.824	55.47 ± 12.30	51.55 ± 14.12	52.73 ± 10.32	0.013
LDL cholesterol (mg/dL)	115.60 ± 32.41	118.80 ± 39.50	118.61 ± 54.10	0.837	125.60 ± 38.19	113.14 ± 44.84	135.67 ± 46.54	0.491
Total cholesterol (mg/dL)	188.75 ± 35.07	192.15 ± 40.56	197.38 ± 45.91	0.079	194.17 ± 35.83	196.35 ± 47.32	236.36 ± 35.77	0.001
Smoker								
No	372 (41.6)	131 (14.7)	52 (5.8)	0.011	887 (85.7)	83 (8.0)	11 (1.1)	0.033
Yes	202 (22.6)	84 (9.4)	53 (5.9)		44 (4.3)	10 (1.0)	-	
Lead level, µg/dL								
0>, <2	436 (48.8)	112 (12.5)	48 (5.4)	0.000	808 (78.1)	71 (6.9)	7(0.7)	0.006
2–<3	115 (12.9)	79 (8.8)	43 (4.8)		105 (10.1)	18 (1.7)	4(0.4)	
3–<4	19 (2.1)	21 (2.3)	10 (1.1)		11 (1.1)	4 (0.4)	-	
≥4	4 (0.4)	3 (0.3)	4 (0.4)		7 (0.7)	-	-	

**Table 2:** Correlation for Sys BP, HDL, Total Cholesterol and Lead levels.

	FRS	BP Sys	_HDL Cholesterol	Total Cholesterol	Leal Level
FRS	1				
BP sys	0.622 (*p* < 0.001)	1			
HDL Cholesterol	−0.185 (*p* < 0.001)	−0.123 (*p* < 0.001)	1		
Total Cholesterol	0.181 (*p* < 0.000)	0.009 (*p* = 0.691)	0.228 (*p* < 0.001)	1	
Lead level	0.441 (*p* < 0.001)	0.204 (*p* < 0.001)	−0.134 (*p* < 0.001)	0.047 (*p* = 0.041)	1

**Table 3:** Odds ratio for FRS among the sub items (Sys BP, HDL, Total Cholesterol) and Lead levels.

10-Year CVD Risk	Category	Male		Female	
		OR (CI)	*p*	OR (CI)	*p*
10–20%	Lead level	2.407 (1.885–3.075)	0.000	1.051 (0.676–1.633)	0.827
	BP sys, mmHg	1.051 (1.037–1.065)	0.000	1.147 (1.120–1.175)	0.000
	HDL cholesterol (mg/dL)	0.991 (0.976–1.006)	0.239	0.987 (0.964–1.011)	0.293
	Total cholesterol (mg/dL)	1.003 (0.999–1.008)	0.172	1.007 (1.000–1.015)	0.066
>20%	Lead level	2.847 (2.020–4.011)	0.000	0.706 (0.188–2.659)	0.706
	BP sys, mmHg	1.121 (1.099–1.143)	0.000	1.220 (1.163–1.281)	0.000
	HDL cholesterol (mg/dL)	0.981 (0.959–1.003)	0.084	0.969 (0.913–1.028)	0.297
	Total cholesterol (mg/dL)	1.009 (1.002–1.015)	0.009	1.041 (1.020–1.063)	0.000

The reference category is: 10-year CVD Risk < 10%.

## Data Availability

Not available.
